# The Effect of Phospholipids (Surfactant) on Adhesion and Biomechanical Properties of Tendon: A Rat Achilles Tendon Repair Model

**DOI:** 10.1155/2015/689314

**Published:** 2015-05-25

**Authors:** T. Kursat Dabak, Omer Sertkaya, Nuray Acar, B. Ozgur Donmez, Ismail Ustunel

**Affiliations:** ^1^Department of Orthopaedic and Traumatology, Akdeniz University School of Medicine, 07059 Antalya, Turkey; ^2^Orthopaedic and Traumatology Clinic, Antalya Training and Research Hospital, 07100 Antalya, Turkey; ^3^Department of Histology and Embryology, Akdeniz University School of Medicine, 07059 Antalya, Turkey; ^4^Department of Nutrition and Dietetics, Akdeniz University Antalya School of Health, 07058 Antalya, Turkey

## Abstract

Adhesion of the tendon is a major challenge for the orthopedic surgeon during tendon repair. Manipulation of biological environment is one of the concepts to prevent adhesion. Lots of biochemicals have been studied for this purpose. We aimed to determine the effect of phospholipids on adhesion and biomechanical properties of tendon in an animal tendon repair model. Seventy-two Wistar rats were divided into 4 groups. Achilles tendons of rats were cut and repaired. Phospholipids were applied at two different dosages. Tendon adhesion was determined histopathologically and biomechanical test was performed. At macroscopic evaluation of adhesion, there are statistically significant differences between multiple-dose phospholipid injection group and Control group and also hyaluronic acid group and Control group (*p* < 0.008). At microscopic evaluation of adhesion, there was no statistically significant difference (*p* > 0.008). Ultimate strength was highest at hyaluronic acid injection group and lowest at multiple-dose phospholipid injection group. Single-dose phospholipids (surfactant) application may have a beneficial effect on the tendon adhesion. Although multiple applications of phospholipids seem the most effective regime to reduce the tendon adhesion among groups, it deteriorated the biomechanical properties of tendon.

## 1. Introduction

Adhesion of the tendon is one of the major challenges for the orthopedic surgeon during tendon repair [1]. In the present day, there is no ideal method to prevent adhesion. Although improvements at the repairmen techniques and rehabilitation have decreased the adhesion rates in tendon surgery, a considerable rate of adhesion still remains. That is why further attempts still continue and one of them is manipulation of biological environment. Amiel et al. showed the beneficial effect of hyaluronan on adhesion after tendon repair [2]. On the contrary, Tuncay et al. reported that there was not any significant effect of hyaluronic acid on adhesion after Achilles tendon repair [3]. 5-Fluorouracil, low molecular weight heparin, amniotic fluid, lubricin, and parecoxib were some of substances reported in the literature in respect to their effects on adhesion after tendon repair [4–8].

Phospholipids, which are the major component of biomembranes, have been determined at the lung as a complex structure, called surfactant, which prevent collapse of alveoli. Later, phospholipids were also found at different body parts like synovial joint, tendon sheet where the friction persists [9, 10]. They were thought to function at the lubrication of the gliding surfaces. There are some studies that examined their role in the friction of tendon in the literature. But there is little information about the effect of phospholipids on the tendon healing and tendon adhesion [11]. In the present study, we showed the effect of phospholipids (surfactant) on the tendon adhesion and biomechanical properties of tendon in rat Achilles tendon healing model.

## 2. Materials and Methods

### 2.1. Animals

Seventy-two Wistar rats were used in the study. Animals were obtained from Akdeniz University Department of Experimental Animals Care and Production Unit and approval was obtained from the University Committee on Animal Resources. Animals were exposed to a 12 h light and 12 h dark cycle at 22°C room temperature. Animals had access to standard laboratory chow and water ad libitum. Experimental procedures were approved and carried out in accordance with Akdeniz University Animal Care and Use Committee's guidelines.

Animals were divided into 4 groups randomly: single-dose phospholipid injection group: after surgery 10 mg phospholipids injection was applied to area between Achilles tendon and skin; multiple-dose phospholipid injection group: after surgery and at 2, 4, 6, and 8 days, 10 mg phospholipids injections were applied to the same area; hyaluronic acid injection group: after wound closure, 3 mg hyaluronic acid was applied to the same area; and Control group: after wound closure 1 mL saline solution was injected in the same area.

Phospholipids (surfactant) were obtained from Professor Dr. Murat Dabak (Internal Medicine Department of Veterinary Faculty of Fırat University, Elazığ, Turkey) [12].

The used hyaluronic acid was Jonexa (Genzyme Corp., USA).

### 2.2. Surgical Technique

Animals were anesthetized by intraperitoneal injection of ketamine hydrochloride (Ketalar, Pfizer-Eczacıbaşı Inc., Turkey) plus xylazine (Alfazyne, Egevet Inc., Turkey) (50 mg/kg + 5 mg). The leg was shaved and prepped with iodine. Then, Achilles tendon was exposed from its origin at the gastrocnemius muscle down to its insertion into the calcaneus through a longitudinal sharp incision. Achilles tendons were transected at 5 mm above its insertion into the calcaneus. The tendon was repaired with 5.0 absorbable suture (Maxon, Coividien Inc., USA) by modified Kessler technique; skin was sutured with 3.0 polypropylene (Propilen, Doğsan Inc., Turkey). After operation animals have been allowed to move freely.

On 28th day, all animals were euthanized. Tendon adhesion (macroscopic and microscopic) was evaluated due to the criteria defined by Tang et al. [13] (Tables 1(a)-1(b)). Macroscopic evaluation of adhesion was made while Achilles tendon sample was taken. Then, Achilles tendons were taken totally by cutting from triceps surae muscle and calcaneus. From each group, six samples have been used for microscopic evaluation and twelve samples for biomechanical test. Biomechanical test was performed by the computerized Lloyd testing machine (Lloyd LR5K, West Sussex, UK) and analyzed by the Lloyd software. The specimens were mounted vertically in the machine with a fixture and biomechanical test was conducted by a 5 kN load cell at a crosshead speed of 2 mm/min at room temperature. During the test, isotonic solution was regularly applied to prevent the tendons from drying. Tensile test was applied until a rupture occurred. At the end of the biomechanical tests, ultimate strength, Young's modulus, and energy absorption capacity of tendons were calculated [14].

### 2.3. Statistical Analysis

Statistical analysis was performed by using SPSS 18.0 software. Kruskal-Wallis test and Bonfferroni test as post hoc were used. Bonferroni correction is used to reduce the chances of obtaining false-positive results since multiple pairwise tests are performed on a single set of data. To perform a Bonferroni correction, the alpha (*α*) was divided by the number of comparisons being made. In our study, there were four groups and six pairwise comparisons. So we divided the alpha by six. The statistical significance level for comparison was set as *p* < 0.008.

## 3. Results

### 3.1. Macroscopic Evaluation of Adhesion

There was more or less adhesion in all groups. Single-dose phospholipid injection group (total 17 samples) had 2 slight adhesions, 14 moderate adhesions, and one severe adhesion. Multiple-dose phospholipid injection group (total 18 samples) had 9 slight adhesions and 9 moderate adhesions and there was no severe adhesion. Hyaluronic acid group (total 17 samples) had 7 slight adhesions and 10 moderate adhesions and there was not any severe adhesion in this group, either. Control group (total 18 samples) had 2 slight adhesions, 9 moderate adhesions, and 7 severe adhesions. There are statistically significant differences between multiple-dose phospholipid injection group and Control group and also hyaluronic acid group and Control group (*p* < 0.008) (Table 2).

### 3.2. Microscopic Evaluation of Adhesion

Single-dose phospholipid injection group had 1 slight adhesion and 5 moderate adhesions and there was no severe adhesion. Multiple-dose phospholipid injection group had 2 slight adhesions and 4 moderate adhesions and there was not any severe adhesion. Hyaluronic acid group had 1 slight adhesion, 4 moderate adhesions, and 1 severe adhesion. Control group had 1 slight adhesion, 2 moderate adhesions, and 3 severe adhesions. When the groups have been compared, there was no statistically significant difference (*p* > 0.008) (Table 2).

### 3.3. Biomechanical Evaluation

**Figure 1:**
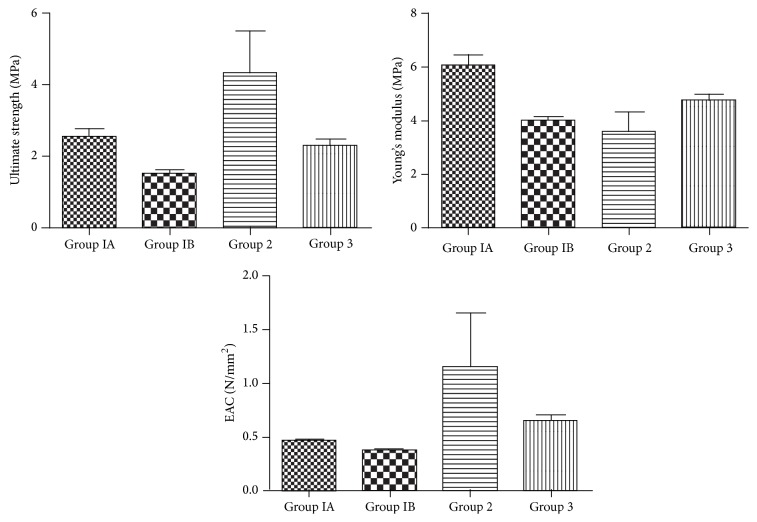
Biomechanical test results: Group IA: single-dose phospholipid injection group; Group IB: multiple-dose phospholipid injection group; Group 2: hyaluronic acid injection group; and Group 3: Control group.

At biomechanical examination, we evaluated Young's modulus, ultimate strength, and energy absorption capacity of tendons (Figure 1). Young' modulus was highest in single-dose phospholipid injection group and lowest in multiple-dose phospholipid injection group. There was statistically significant difference between these groups (*p* < 0.05). Ultimate strength was highest in hyaluronic acid injection group and lowest in multiple-dose phospholipid injection group. However, there was not any statistically significant difference (*p* > 0.05). Energy absorption capacity was highest in Control group and lowest in multiple-dose phospholipid injection group. But there was no statistically significant difference between two groups (*p* > 0.05).

## 4. Discussion

Adhesion of tendon to surrounding tissues is one of the major complications which have been encountered quite often during tendon repair. Although alterations in tendon suturing techniques and rehabilitation schedules have improved the outcomes, efforts still continue to minimize the adhesion of the healing tendon which prevents movement resulting in poor functional outcomes.

Another point of view for prevention of the adhesion is to modify the environment. For this purpose, lots of different modalities from barriers to biochemical agents have been examined. Hydroxyapatite and alumina sheath, amniotic membrane, polyvinyl alcohol-hydrogel, vein graft, and hydrogel sealant-like materials have been examined as barriers to prevent tendon adhesion [15–19]. The effects of many biochemical agents like amniotic fluid, hyaluronic acid, 5-fluorouracil, low molecular weight heparin, lubricin, and mannose-6-phosphate have also been reported in the literature with varying results [2, 4–8, 20].

Phospholipids, which are known as major component of the biomembranes, were firstly determined to play a crucial role in the lung as surfactant. Later, phospholipids were found at synovial joints as surface active phospholipids (SAPL) [9]. They have been proposed to be the substance responsible for boundary lubrication at the synovial joints. However, this issue is still controversial since some other substances like lubricin have also been propounded to be responsible for boundary lubrication. Afterwards, Mills et al. have shown phospholipids at tendon and tendon sheath of equine tendon [10]. They reported SAPL as a multilayered lining on the epithelial surface of the flexor tendon and the inner tendon sheath. They also determined that SAPL concentration in the tendon sheath fluid is much lower than synovial joints and said that it was because of greater direct load bearing in the joints compared to tendons. However, the scope of this research was not on the tendon healing and/or adhesion.

Wherever the surfactant was determined in the body, its composition was shown to differ in terms of proportions and types of phospholipids. Chen showed that unsaturated phospholipids were predominant at the bovine synovial joint [21]. On the other hand, predominant phospholipid in the lung surfactant is phosphatidylcholine which is a saturated phospholipid [22]. Mills et al. determined the phospholipids on the tendon and tendon sheath but did not classify the subgroups [10]. And also we could not find any other information about the subgroups of phospholipids at the tendon and tendon sheath.

Moro-oka et al. studied the effect of dipalmitoyl phosphatidylcholine and hyaluronic acid mixture on the tendon adhesion and reported affirmative effects of this mixture on prevention of tendon adhesion after tendon injury [11]. However, from lung surfactant, we know that not a single phospholipid but a mixture of them functions at lung. Additionally, there are some other molecules like apoproteins which play role in the regulation of surfactant function at lung [22]. In addition, we also know that different types of phospholipids were shown to present at the synovial joints [21]. That is why we plan to use phospholipids in composite form in our study. Since the lung surfactant (which contains different types of saturated and unsaturated phospholipids) is the unique natural source of such a mixture in practice, we preferred to use lung surfactant in this study. Sun et al. tested the changes in the friction of the canine flexor tendon treated with lipid solvents and phospholipase C [23]. They reported that the increase in the friction suggested the presence of phospholipids on the tendon. They also determined that a more distinct increase in friction by lipid solvents compared to phospholipase C (which selectively removes certain types of phospholipids like phosphatidylcholine) suggested the presence of different types of phospholipids on the tendon. This result fortifies our hypothesis.

In our study, there was more or less adhesion in all study groups at macroscopic evaluation of adhesion. However, there was not any severe adhesion in multiple-dose phospholipid injection group and hyaluronic acid group and also the lowest rate of adhesion has been determined in these groups. The highest rate of adhesion was in Control group. There were statistically significant differences between multiple-dose phospholipid injection groupand Control group and hyaluronic acid group and Control group. On the other hand, there was not statistical difference between multiple-dose phospholipid injection group and hyaluronic acid injection group. Although the result of single-dose phospholipid injection group was better than Control group in terms of macroscopic adhesion, there was no statistically significant difference.

Results of single-dose phospholipid injection group, multiple-dose phospholipid injection group, and hyaluronic acid group were similar in respect to microscopic evaluation of adhesion. Nevertheless, it was noticed that there was no severe adhesion in single-dose phospholipid injection group and multiple-dose phospholipid injection group. Although more adhesions were determined in the Control group, there was not any statistically significant difference between groups. Moro-oka reported that dipalmitoyl phosphatidylcholine and hyaluronic acid mixture decreased the tendon adhesion [11]. ArRajab et al. reported that exogenous phospholipid caused a moderate reduction on postoperative peritoneal adhesion in rats [24]. Bhandarkar determined that spraying DPPC : PG on the environment was a more potent way of phospholipid application to reduce peritoneal adhesion in rabbits [25]. Amiel et al. reported that hyaluronan prevented adhesion after tendon repair [2]. Akasaka et al. showed that hyaluronic acid decreased both the adhesion and the resistance during tendon movement [26]. On the contrary, De Wit et al. reported that there was not any effect of hyaluronic acid on the tendon adhesion but there was on healing [27].

In the literature, there was not any information about the effect of the phospholipids on biomechanical properties of tendon after repair. However, there are studies reporting that hyaluronic acid improved the tendon healing [27, 28]. When the biomechanical properties of the tendons were evaluated, the results of single-dose phospholipids injection group and the hyaluronic acid injectiongroup were similar. On the contrary, we determined that multiple phospholipids applications deteriorated the biomechanical properties of tendons. Hills reported that fibroblast sticking could be neglected by SAPL preabsorbed into the surface [29]. So, it may be thought that multiple injections of phospholipids impair the fibroblast function during tendon healing while preventing adhesion.

## 5. Conclusion

Single-dose phospholipids (surfactant) application may have a beneficial effect on the tendon adhesion and does not disturb the biomechanical properties of the tendon. On the other hand, although multiple applications of phospholipids seem the most effective regime to reduce the tendon adhesion among groups, it deteriorated the biomechanical properties of tendon.

## Figures and Tables

**(a):** 

Points	Features of adhesion
Quantity	
0	No apparent adhesions
1	A number of scattered filaments
2	A large number of filaments
3	Countless filaments

Quality	
0	No apparent adhesions
1	Regular, elongated, fine, and filamentous
2	Irregular, mixed, shortened, and filamentous
3	Dense, not filamentous

Grading of adhesions	
0	None
1-2	Slight
3-4	Moderate
5-6	Severe

**(b):** 

	Points	Adhesion appearance
Length	0	No adhesion
	1	Localized, <10 mm longitudinal
	2	10–15 mm
	3	Intense, >15 mm

Characteristics	0	No adhesion
	1	Loose, elastic, and mobile
	2	Of average thickness and mobile
	3	Thick, hard, and immobile

Grading	0	No adhesion
	2	Mild adhesion
	3-4	Moderate adhesion
	5-6	Advanced stage adhesion

**Table 2:** Rates of macroscopic and microscopic adhesion of groups according to Tang criteria.

	Adhesion (%)							
	Macroscopic adhesion (*n* = 70)				Microscopic adhesion (*n* = 24)			
	None	Slight	Moderate	Severe	None	Slight	Moderate	Severe
Single-dose phospholipid injection group	0	11.80	82.40	5.80	0	16.70	83.30	0
Multiple-dose phospholipid injection group	0	50.0	50.00	0.00	0	33.30	66.70	0
Hyaluronic acid group	0	41.20	58.80	0.00	0	16.70	66.70	16.70
Control group	0	11.10	50.00	38.90	0	16.70	33.30	50.00
